# Virus-Bacteria Interactions: An Emerging Topic in Human Infection

**DOI:** 10.3390/v9030058

**Published:** 2017-03-21

**Authors:** Erin A. Almand, Matthew D. Moore, Lee-Ann Jaykus

**Affiliations:** 1 Department of Microbiology, North Carolina State University, Raleigh, NC 27695, USA; eaalmand@ncsu.edu (E.A.A.); lajaykus@ncsu.edu (L.-A.J.); 2 Department of Food, Bioprocessing and Nutrition Sciences, North Carolina State University, Raleigh, NC 27695, USA

**Keywords:** interaction, enteric, respiratory, bacteria, virus, pathogenesis, bacteria-virus interaction

## Abstract

Bacteria and viruses often occupy the same niches, however, interest in their potential collaboration in promoting wellness or disease states has only recently gained traction. While the interaction of some bacteria and viruses is well characterized (e.g., influenza virus), researchers are typically more interested in the location of the infection than the manner of cooperation. There are two overarching types of bacterial-virus disease causing interactions: direct interactions that in some way aid the viruses, and indirect interactions aiding bacteria. The virus-promoting direct interactions occur when the virus exploits a bacterial component to facilitate penetration into the host cell. Conversely, indirect interactions result in increased bacterial pathogenesis as a consequence of viral infection. Enteric viruses mainly utilize the direct pathway, while respiratory viruses largely affect bacteria in an indirect fashion. This review focuses on some key examples of how virus-bacteria interactions impact the infection process across the two organ systems, and provides evidence supporting this as an emerging theme in infectious disease.

## 1. Introduction

Although commensal bacteria colonize a variety of body systems, the respiratory and gastrointestinal microflora are the most widely studied. Gastrointestinal habitats incorporate anywhere from 200 species (within the oral cavity) to 1000 species at the distal intestine, where bacterial concentrations can reach 10^14^ cells/g. Conversely, respiratory tracts contain only about 10^4^ total bacteria and ample uninhabited space. A wide variety of both commensal and pathogenic organisms colonize the nasopharynx, causing infections in both the lower respiratory tract and upper respiratory tract when host homeostasis is compromised [1,2,3,4].

The composition and cell concentrations in these microbial populations mediate intimate interactions between the host and commensal bacteria. Previous reviews have provided a great detail on the mechanisms of virus-bacterial interactions occurring either within the upper respiratory or gastrointestinal tracts [5,6,7,8,9]; however, these similarities and differences are observed in isolation, rather than highlighting the interactions across organ systems. The purpose of this brief review is to examine virus-bacteria interactions that result in enhanced pathogenesis in these two organ systems. It should be noted that bacteria-virus interactions are complex, and there is much that is still unknown. The scope of this brief review will only include some of the common themes in bacteria-virus interaction that result in enhanced pathogenesis, as numerous reports have documented bacterial inhibition of viral infection as well [10,11,12].

Categorically, bacteria and viruses interact in two ways (Table 1). The mechanism(s) benefitting virus infiltration predominately occur(s) through direct interactions, by (i) viral binding to a bacterial cell, or (ii) viral utilization of a bacterial product. These interactions promote infection of the virus, with no known benefit to the bacterial species. Conversely, bacterial advantages are usually gained through indirect interactions in which the virus inflicts host cell damage critical to virus infection, but is in turn beneficial to other pathogens. In these instances, there is no direct physical interaction between the virus and the bacteria; rather, the viral infection makes one or more host cell types more susceptible to bacterial colonization. There are four major mechanisms, often working in concert, supporting the indirect interactions: (i) virus-induced increase of bacterial cell receptor concentrations; (ii) virus damage to underlying epithelial cells; (iii) virus displacement of commensal bacteria; and (iv) virus suppression of the host immune system.

## 2. Direct Interactions: Viruses Exploiting Bacteria

The majority of reported direct bacteria-virus interactions are associated with viruses infecting the gastrointestinal tract. In this body system, commensal bacteria are considered the first line of defense against invading pathogens by outcompeting their disease-promoting counterparts and limiting tissue accessibility. Undoubtedly, enteric viruses encounter these large numbers of diverse commensal bacteria, but rather than always preventing infection, some viruses evolved to exploit this contact, facilitating the disease process [8].

Under in vitro conditions, viruses may be able to directly bind to their target cell type and undergo replication with ease. However, this strategy may prove problematic in the gastrointestinal tract where a large number of bacteria occupy tissue surfaces, directly competing for receptor binding sites, and reducing the likelihood of pathogenic bacterial proliferation or virus attachment. Other components, like mucus or enzymatic secretions, may also interfere or assist the infection process. To circumvent this, rather than compete for host cell binding sites, some viruses can utilize bacterial ligands to enhance their association with more accessible host cells, initiating infection. This same strategy may be employed by some viruses that may not exclusively target the host’s epithelial cells and use bacteria to assist infection of other cell types in addition to or exclusive of epithelial cells [42,43].

An increasing body of work highlights how certain types of bacteria promote viral disease symptoms, suggesting bacterial populations may aid infection [9]. As research into this area expands, specific bacterial targets are being identified as viral binding sites. Certain enteric viruses illustrate how a bacterial component—attached or independent to the bacterium itself—promotes the virus infection cycle. Poliovirus, which replicates in the intestine prior to systemic dissemination, is a good example [43]. In a comprehensive study [18], mice with and without normal gut microflora were orally challenged with poliovirus. The former group demonstrated mortality twice that of the mice treated with antibiotics. This increase in mortality was associated with increased viral titers in mice with the intact microbiota. These effects were not observed after intraperitoneal challenge in which the virus does not need to interact with the native microbiota prior to infection. When these findings were examined using a cell culture model, exposure of poliovirus to bacteria or bacterial components increased virus titers as much as 500% and doubled the poliovirus adherence to HeLa cells [18]. It was hypothesized that bacterial components—lipopolysaccharides (LPS), peptidoglycan, and other *N*-acetylglucosamine-containing polysaccharides—increase viral receptor binding and increase viral shedding [18]. Interestingly, evidence has been reported that this also may be the case for murine norovirus, as antibiotic-treated mice show reduced virus titers compared to mice having normal gut microflora [10,39]. A similar mechanism may also be used by human norovirus. Histo-blood group antigens (HBGAs) have been identified as putative host cell receptors or co-receptors for this virus, and HBGA-like moieties have been reported to be present on the surface of some enteric bacteria (i.e., *Enterobacter cloacae*) [14,44,45]. These motifs were found in the bacterial extracellular polymeric material and bound to representative human norovirus strains. Furthermore, bacterial components have been reported to facilitate viral replication in a BJAB cell culture system. More specifically, the bacterial derived HBGA-like molecules stimulated replication comparable to their synthetic counterparts, in a dose dependent manner for *Enterobacter cloacae*, a commensal enteric bacteria. Further, the data presented suggests bacterial HBGAs in *Enterobacter cloacae* enhance norovirus attachment to target cells, increasing viral infectivity [13]. Expanding on these results, a recent study suggests that some commensal *Escherichia coli* strains expressing HBGA-like moieties may aid norovirus resistance to heat [45], which may have implications for norovirus persistence, however, future study of bacterial effect on viral persistence must be conducted. Interestingly, this is a similar phenomenon to that which was observed with poliovirus, as binding to bacteria also increased the stability of the viral capsid when exposed to heat [19]. Thus, both poliovirus and norovirus provide examples of viruses with enhanced pathogenesis when directly binding commensal enteric bacteria.

Although less prominent in the literature, members of the *Reoviridae* family of viruses may also exhibit similar gut microbiota interactions. In one study, a cohort of mice was treated with antibiotics prior to challenge, and disease pathology was compared to untreated but challenged animals to test the contribution of the native gut microflora. The untreated mice had classic reovirus strain T3SA+ symptoms with biliary obstructions and enlarged Peyer’s patches. The antibiotic-treated mice appeared normal, and also had significantly lower reovirus titers in the intestine. Similarly, poliovirus infection was enhanced in vivo in a mouse model with wild type mice versus germ-free or antibiotic-treated mice. Additional work also showed that the presence of bacteria enhanced poliovirus replication using a plaque assay [18]. Like norovirus, this may be related to bacteria presenting carbohydrates, since rotavirus and reovirus TS3A+, different genera in the *Reoviridae* family, exploit HBGAs and sialic acid carbohydrates as cellular receptors, respectively [46,47]. However, the nature of these interactions is poorly characterized, as is the role of HBGA or HBGA-like moiety binding in the rotavirus infection process [20]. Certainly, future work describing the specific mechanisms for the enhancement of reovirus and rotavirus infectivity is forthcoming.

In addition to binding and stabilization of viral capsids, bacterially synthesized enzymes can also stimulate viral infection. In this instance, bacterial presence not only increases influenza virus adhesion [48], but the virus gains a foothold by utilizing bacteria components for infection. To become infectious, the precursor hemagglutinin (HA_0_) of influenza needs to undergo proteolytic cleavage into HA_1_ and HA_2_ fragments. Typically, the host supplies enzymes necessary for activation; however, studies also implicate proteases produced by *Staphylococcus aureus* and *Aerococcus viridans*. Such synergism therefore promotes viral pathogenesis [21,22,23]. Unlike the previous examples where enhanced viral pathogenesis likely stemmed from viruses binding the bacteria, this is an example of viruses enhancing pathogenesis by also directly utilizing a bacterial product to aid infection.

Human immunodeficiency virus (HIV) provides a unique example of the interdependence between virus and bacteria. While previous examples dealt with commensal bacteria exploited for viral invasion, HIV recruits another human pathogen. More specifically, some evidence exists that individuals infected with HIV are more prone to *Mycobacterium tuberculosis*, and infection with the bacteria accelerates the progression of the acquired immune deficiency syndrome (AIDS). Latent infection of individuals with *M. tuberculosis* has been reported, and in some cases “reactivation” occurs where disease symptoms are observed years after initial infection [49]. Multiple specific mechanisms of HIV promotion of *M. tuberculosis* reactivation have been reported; for example, depletion of CD4^+^ T cells and up-regulation of the CD14 (which may aid *M. tuberculosis* infection) in macrophages, among other proposed mechanisms (reviewed in [25]). During acute *M. tuberculosis* infection, HIV RNA copy number increases [24,25], possibly due to the interplay between the *M. tuberculosis* cell wall component lipoarabinomannan and the immune system. *M. tuberculosis* up-regulates the production of tumor necrosis factor (TNF), an immune system component that controls bacterial infections, which activates HIV replication in macrophages [25]. The immune system also produces interleukin (IL)-6 which, in conjunction with higher TNF, activates transcription of the long terminal repeats in HIV, abetting replication [50]. The immune system is also involved in the bacteria-virus interactions of another retrovirus, mouse mammary tumor virus (MMTV), which interacts with commensal enteric bacteria. In this case, evidence was reported suggesting that MMTV binds enteric bacterial LPS that initiates Toll-like receptor 4 (TLR4, a pattern recognition sensor that targets LPS) that then activates IL-10 and then IL-6 with the effect of allowing the MMTV antigen to evade the immune response and persist in the host. In other words, MMTV binds to and uses bacterial LPS to “cloak” itself from the immune system and persist [26,27]. Both MMTV and HIV are examples of viruses exploiting bacterial effects on the immune system for enhancing infection.

Although the nature of the interaction remains the same, there may be additional benefits to viruses with bacteria interactions other than direct disease progression. Studies have also shown that association with fecal microbiota increased poliovirus environmental fitness and stability, as exposure to bacteria or their polysaccharides decreased the efficacy of virus inactivation by heat and bleach, potentially aiding viral survival in the environment [19]. This observation was further supported by the higher susceptibility to inactivation with heat observed in a poliovirus mutant that did not bind LPS as efficiently. Furthermore, the introduction of these bacterial polysaccharide components has been found to enhance wild type poliovirus binding to its host cells expressing its receptor [19]. In short, gastrointestinal microbiota not only increase poliovirus infectivity, but may also promote virus transfer to the next host. Thus, there are numerous ways that direct viral interaction with bacteria aid viral pathogenesis, and this topic is an emerging area of study in microbiology.

## 3. Indirect Interactions: Bacteria Exploiting Viral Infections

Bacterial species often benefit from viral infections. Although the virus exists independent of the proximal bacterial species, the virus-induced disease state can allow normally harmless bacteria to become opportunistically pathogenic. Under normal, healthy circumstances, direct competition between microbes limits pathogen invasion by saturating colonization sites, priming barrier immunity to produce antimicrobials, and increasing the immune response to invading microorganisms [51]. When microbial populations are disrupted, niches previously inaccessible to invading pathogens become available, and surfaces where native microbiota previously outcompeted their disease-causing counterparts are compromised. The overall ways viruses aid bacteria pathogenesis include a complex combination of cellular receptor up-regulation, disruption of the epithelial layers, displacement of commensal bacteria, and immune system suppression. Arguably, the interactions between influenza viruses and pathogenic bacteria (i.e., *Streptococcus pneumoniae*, *S. aureus*, and *Haemophilus influenzae*) remain the best studied within the human body, and they exemplify some of these mechanisms with both viruses and bacteria benefitting from the relationship. Influenza viruses not only damage the host epithelium (e.g., apoptosis), they also provide potential binding sites for bacteria through three mechanisms: neuraminidase cleavage of sialic acid from host cells, bacterial host receptor up-regulation, and host regeneration of the common bacterial receptors fibrin and fibrinogen [38,49,52]. This pattern of host damage is common amongst upper respiratory tract viruses and bacteria (reviewed in [5,6,7,53]), and these interactions are summarized in Table 1. Thus, these numerous dynamics that exist between influenza and bacteria exemplify the multiple complex ways viral infection can indirectly assist bacterial infection.

Another type of virus-bacteria interaction of interest is immune system subversion, which occurs when viruses target cell types such as lymphocytes, macrophages, and monocytes. By infecting and replicating within cells originally primed for defense, the host response is severely hindered (for a thorough review of the immune system subversion of respiratory or enteric viruses see [6] or [9], respectively). Once again, influenza has also been documented to promote this type of dynamic as well, as the viruses deplete alveolar macrophages, impairing bacterial clearance of the pathogenic *S. pneumonia* [54,55]. Furthermore, the virus alters the Toll-like receptor pathways, resulting in decreased neutrophil attraction, which in turn increases the attachment of bacterial cells onto host epithelium [7]. Additionally, infection with influenza can deregulate cytokine production through inducing type I interferon, which can down-regulate cytokine production [56]. MicroRNA-based mechanisms resulting from influenza infection can also down-regulate cytokine production. Specifically, influenza infection was found to up-regulate microRNA-155 in macrophages, which has been found to down-regulate IL-17 and make the host more susceptible to bacterial infection [57]. Another classic example of immune subversion is found in the periodontal diseases gingivitis and periodontitis [58]. Research shows that the more aggressive periodontitis (associated with attachment, bone, and tooth loss) is the result of interactions between three herpes virus species (Epstein-Barr virus type 1 (EBV-1), human cytomegalovirus (HCMV), and herpes simplex virus (HSV)) with the common periodontal bacteria *Porphyromonas gingivalis* and *Dialister pneumosinte* [59,60]. The collaboration between virus and bacteria is two-fold: through an impaired immune system and lesion development [28]. HSV and HCMV both infect monocytes, macrophages, and T-lymphocytes, whereas EBV-1 targets B-lymphocytes. Virally infected immune cells cause inflammation and cytopathic effects within host tissues, while providing a diminished capacity to defend against periodontal bacteria. This inflammation provides the starting point for periodontal lesions. Furthermore, the viral proteins present on these infected but intact host cells act as receptors for periodontal bacteria, while destroyed host cells also provide attachment points at newly exposed surfaces [59]. These lesions progress until the rapid loss of connective tissue attachment and alveolar bone loss characteristic of periodontitis occurs. Thus, immune system subversion is a dynamic that must be considered when understanding virus-bacteria dynamics with regards to pathogenesis.

The interactions mentioned thus far provide localized instances where viruses and bacteria aid each other to infiltrate surrounding tissues. Some viruses, however, not only act on one location within the body, but rather directly impact the immune system, allowing for bacterial co-infections to arise separately from the viruses’ area of impact. For instance, HIV targets helper T lymphocytes, macrophages, and dendritic cells. By targeting a wide variety of cells within the immune system, its pathogenesis promotes highly complex polymicrobial interactions characterized by bacterial co-infections within the entire human microbiome [61]. The gastrointestinal tract bears the brunt of the attack, where the virus contributes to increased bacterial translocations, injured immune system components (i.e., anti-inflammatory and pathogen recognition pathways), and depleted commensal flora. Additionally, HIV immune system disruption changes the overall composition of certain host microbiomes, causing once static bacterial ratios in the oral cavity and gastrointestinal tract to diversify with new inhabitants [32]. This phenomenon contributes to hallmark symptoms such as oral lesions, chronic lung disease, and pneumonia [32,62]. While the exact mechanisms of these effects are unknown, depletion of immune cells commonly found at highly populated host-bacteria interfaces leads to barrier breakdowns and increased bacterial colonization. In some cases, this results in pathogen infiltration, like that observed with increased *M. tuberculosis* infections, but often times the impact may be more subtle. Measles is a persistent virus often recovered from T-lymphocytes, B-lymphocytes, and macrophages, while also permeating the central nervous system and lymph nodes. While measles viruses rarely result in mortality, they increase susceptibility to secondary bacterial infections. Measles viruses suppress the antibacterial responses of both the innate and adaptive immune systems, proving ample opportunities for opportunistic pathogens to invade. Reports associate *Listeria monocytogenes*, *S. aureus*, and *M. tuberculosis* infections with measles viruses, and likely other bacterial agents also capitalize on the immunocompromised host [30,31,63]. One specific example of measles-related immunosuppression occurs when measles viruses bind their receptor human signaling lymphocyte activation molecule (hSLAM) and infect dendritic cells. Such infection down-regulates expression of a number of different surface molecules on the cells, including CD40, which is involved in the proliferation of CD8^+^ T cells. Additionally, dendritic cells are inhibited from differentiating into mature effector dendritic cells by the infection and the rate of apoptosis of these cells were found to be elevated [30]. Thus, the examples listed here provide strong evidence that systemic effects of viral infection on the immune system must be taken into account in relation to potential co-infection of bacteria.

## 4. Influence of Bacteria and Virus Features on Interactions

The interactions described above do not appear to be exclusive to Gram-positive or Gram-negative bacteria. Within the direct interactions category, both types of bacteria (and their LPS and/or peptidoglycan components) stabilize poliovirus, while only Gram-positives aid influenza viruses. Other enteric virus studies have preferentially investigated Gram-negative bacteria or their membrane component (LPS), possibly due to their abundance in the intestines. In the case of norovirus, it is likely that Gram-negative bacterial interactions were studied because of previous research suggesting the presence of HBGA-like antigens on their surfaces, but evidence of norovirus binding to Gram-positive bacteria and the presence of HBGA-like moieties has recently been reported [15]. For the studies observing interactions within the entire gastrointestinal microbiome (e.g., murine norovirus), it is unclear if other bacterial species in addition to *Enterobacter cloacae* support infection. For the indirect interactions, both Gram-negative and Gram-positive bacteria have been reported to be positively affected by the virus interaction(s). This is not surprising, as the virus directly affects the host rather than the bacterium, suggesting a less specific, broader reaching mechanism. Both enveloped and non-enveloped viruses are capable of interacting with commensal or pathogenic bacteria, but the means by which this occurs is virus-specific. Influenza, an enveloped virus, utilizes an exogenous bacterial enzyme to cleave hemagglutinin and render itself infectious. Conversely, the non-enveloped viruses (i.e., norovirus, poliovirus, and reovirus) bind directly to bacterial glycoconjugates. In fact, the use of glycosphingolipids as cellular receptors is actually well documented in both non-enveloped and enveloped viruses [64]. However, the extent to which such viruses attach to the corresponding bacterial rather than host motif requires further examination. Although it should be taken into consideration, the structural features of bacteria should not exclude the possibility that a dynamic exists with a virus where pathogenesis of either could be promoted.

Mechanistically there are differences between the polymicrobial interactions observed in the different host tissues. Within the intestine, the enteric viruses have been more commonly observed in direct interactions with the bacteria (e.g., poliovirus), whereas the infections within the lung have been more commonly reported to capitalize on microbial scarcity (for the initial viral infection) and then cellular damage for the bacteria to gain a foothold (e.g., respiratory syncytial virus). Although there are differences between the types of interactions within each host region, both of them involve manipulating the current host status to the benefit of the organism, rather than hijacking and subverting the other microorganism. While this synergism does not always exist (for more information read [65]), this review highlights multiple examples where bacteria and viruses work together to promote pathogenesis. Therefore, dynamics between viruses and bacteria should be a consideration in understanding pathogenesis.

## 5. Conclusions

As our understanding grows, polymicrobial interactions move from the exception to the norm, and researchers must realize viruses and bacteria are no longer mutually exclusive disease-causing agents. While evidence suggests potential antagonistic effects where the bacterial microbiome protects the host from viral infection [66], this review highlights the increased pathogenicity occurring as a consequence of virus-bacteria interactions in areas inhabited by normally benign members of the native microflora. Viruses are utilizing bacterial components to enter target cells, while bacteria capitalize on the destructive nature of virus replication to gain footholds into previously inaccessible regions. Throughout the body these microorganisms can collaborate to better each other, to the detriment of the host. Further elucidation and discovery of virus-bacteria relationships and mechanisms involved in infection is crucial. Although technically challenging, such advancement may require development or improvement of new in vitro or in vivo models. Certainly, advances in metagenomics and the microbiome will play an important role in better understanding these environments and interactions. By focusing on microbial interactions instead of solely on the causative disease agent, it may be possible to exploit these pathways in an effort to identify new therapeutic targets.

## Figures and Tables

**Table 1:** Virus-bacteria interactions. Human viruses often directly and indirectly interact with bacteria. Direct interactions involve a specific bacterium or bacterial product that aids viral infection. Indirect partnerships are the result of a primary viral infection producing amenable conditions for bacterial colonization.

Virus	Bacteria	Significance	Reference
**Direct Interaction**			
Human norovirus	*Enterobacter cloacae*	Histo-blood group antigen (HBGA)-like moieties serve as co-factor during infection	[13,14,15,16]
Murine norovirus	*E. cloacae*, enteric bacteria	HBGA-like moieties serve as co-factor during infection; evidence of the presence of intestinal microbiota aid establishment of persistent viral infection	[13,17]
Poliovirus	*N*-acetyl glucosamine containing polysaccharides (lipopolysaccharide, peptidoglycan)	Enhanced cell association and viral replication; increased capsid stability and transmission	[18,19]
Reovirus T3SA+	Enteric bacteria; *Escherichia coli*, *Ochrobactrum intermedium*, *Bacillus cereus*, *Enterococcus faecalis* (LPS)	Enhanced viral replication; enhanced virus binding/entry	[18]
Rotavirus	Enteric bacteria	Enhanced viral replication; enhanced virus binding/entry; less effective host antibody response	[20]
Influenza virus	*Staphylococcus aureus*; *Aerococcus viridans*	Protease cleaves the hemagglutinin (HA) into HA1 and HA2, making the particles infectious	[21,22,23]
Human immunodeficiency virus (HIV)	*Mycobacterium tuberculosis*	Increases HIV long terminal repeat-driven transcription and HIV production	[24,25]
Mouse mammary tumor virus (MMTV)	Enteric bacteria, *Escherichia coli* EH100, *E. coli* O26, *E. coli* O55:B5, *Bacillus thetaiotaomicron*, *Rhodobacter sphaeroides*, extracted bacterial lipopolysaccharides (LPS)	Virus contains factors on outer membrane that bind bacterial LPS; Uses LPS to promote a Toll-like receptor 4 (TLR4) response that helps it evade host immune system.	[26,27]
**Indirect Interaction**			
Herpesviruses	*Porphyromonas gingivalis*; *Dialister pneumosintes*	Promotes immunosuppression leading to bacterial colonization	[28,29]
Measles virus	*M. tuberculosis*; *S. aureus*; *Listeria monocytogenes*	Promotes a generalized state of immunosuppression leading to bacterial co-infection	[30,31]
HIV	Oral, gastrointestinal, lung, penile, vaginal bacteria	Immune system deterioration and increased bacterial translocation	[32]
Parainfluenza virus	Nasopharyngeal bacteria	Increased bacterial binding to the lower respiratory tract	[33,34]
Respiratory syncytial virus	*Streptococcus pneumonia*, *Pseudomonas aeruginosa, Haemophilus influenzae*	Increased bacterial invasiveness; increased host cell adhesion molecules	[35,36,37]
Influenza virus	*Streptococcus pneumoniae*; *S. aureus*; *H. influenza*; respiratory commensals	Viral neuraminidase cleaves epithelial cell sialic acid exposing bacterial receptors; damages epithelial cells	[6,21,38]
Rhinovirus	*S. pneumoniae*; *S. aureus*; *H. influenzae*	Increases host cell adhesion molecules	[39]
Adenovirus	*S. pneumoniae*	Increases host cell adhesion molecules	[40,41]
